# Pediatric Allergy

**DOI:** 10.1155/2012/414762

**Published:** 2012-05-20

**Authors:** Mary Beth Hogan, Jacqueline A. Pongracic

**Affiliations:** ^1^Department of Pediatrics, University of Nevada School of Medicine, Reno, NV, USA; ^2^Department of Pediatrics, Northwestern University Feinberg School of Medicine, and Children's Memorial Hospital, Chicago, IL, USA

Allergist/immunologists who treat children are often left to apply clinical solutions that were developed in adult populations. Pediatric allergists intuitively know that differences in disease between adults and children have widely divergent contributory factors. The inception of allergic sensitization followed by the development of diseases such as atopic dermatitis and asthma suggests a different treatment paradigm than adult diseases in which inflammation is typically long standing and may possibly be irreversible. In fact, pediatric clinicians are focused on (1) treatments specifically designed for children, (2) treatments which may prevent allergic sensitization, and (3) the prevention of disease progression. Research that is focused upon these objectives is uniquely positioned to advance the understanding and treatment of pediatric allergic diseases.

There are other factors which have adversely affected progress in this area. New investigators who are trained in pediatrics are difficult to find. This is a significant problem in the particularly small field of pediatric allergy/immunology. The discipline is in the process of establishing a significant presence in general pediatric training programs. This special issue is designed to showcase pediatric allergy/immunology investigators. The research articles are focused on identifying elements surrounding the onset of atopy or therapies designed for children. The review articles are aimed at new and provocative thinking regarding the development of atopy. This issue unites pediatric allergists worldwide in establishing a forum of discussion around the issues of atopic sensitization in children and the treatment of these diseases.

New clinically relevant research in pediatric allergy is vital to our field. M. Ben-Shoshan et al. report on demographic factors as predictors of development of food allergy. This work could identify which populations should be targeted for prevention, education, and therapeutic strategies in the future. A. Gangemi et al. present a provocative preliminary study outlining a possible role of L-carnitine in the treatment of pediatric asthma. Their findings could lead to investigation of alternative treatments for asthma in children. Other investigators have focused on the therapy of Hymenoptera venom anaphylaxis with an ultrarush protocol. Venom allergy, like asthma, is clearly different in children than adults. The establishment of the safety of this protocol advances the care of children with this potentially life-threatening disease. In addition, the effect of regional pollen exposure upon the development of aeroallergen sensitization is a practical reminder to pediatric allergists of the origins of allergic rhinitis and asthma. This study illustrates the importance of understanding the changing aeroallergen environment in which children live and in which pediatric allergists practice. 

Immune responsiveness and allergy are integrally related and multiple organ systems can be affected, even in childhood. A review article regarding the potential of a linkage between allergy and immune deficiency takes us back to fundamental elements in immunology. Another factor in the development of atopy is discussed in an article reviewing the role of skin barrier function in the atopic march. Failures of barrier function can increase exposure of the immune system to allergen, thereby potentiating the onset of allergic sensitization. R. J. Hopp reviews pediatric eosinophilic esophagitis and illustrates the complex and progressive nature of this increasingly common allergic disease. As in the case of venom allergy and asthma, eosinophilic esophagitis is different in children versus adults. Clinical studies are lacking in pediatric eosinophilic esophagitis. This article is a siren call to address the initiating factors for this disease and of the need for pediatric-based therapies tailored for the chronicity and complexity of eosinophilic esophagitis. A broad view of the environmental factors that affect exhaled nitric oxide levels in asthmatic children reveals that indoor and outdoor pollutants can affect the inflammatory factors involved in the pulmonary tree. In addition, these triggers may affect the interpretation of obtained levels.

Psychosocial issues in pediatric anaphylaxis are frequently debilitating and hinder effective management. This review article outlines these common problems. Psychosocial influences in pediatric allergic diseases are taken one step further in a provocative review by C. L. Duncan et al. This article suggests that not only do psychosocial factors influence the development and treatment of atopic diseases, but those psychosocial factors might influence the development of atopy via a novel epigenetic route.

This issue revolves around original research in pediatric allergy/immunology and thoughtful review articles in this field. This mixture was designed to discuss hot issues in our understanding of the initiation of pediatric allergic diseases and the treatment of those diseases once established. We hope that clinicians who treat children with allergic diseases will enjoy this blend of exciting new research and reevaluation of the customary way of thinking of these problems. Pediatric allergists interested in food allergy, atopic dermatitis, asthma, and anaphylaxis should continue to question and investigate the many avenues of “Why do children develop allergies?” The fruit of their labors will not only benefit children but also adults. What a wonderful reversal of the usual trend would this be !

*Mary Beth Hogan* *Mary Beth Hogan* *Jacqueline A. Pongracic* *Jacqueline A. Pongracic*

